# Crystal structure and Hirshfeld surface analysis of (*E*)-2-(5-bromo-2-hy­droxy­benzyl­idene)hydrazine­carbo­thio­amide di­methyl sulfoxide monosolvate

**DOI:** 10.1107/S2056989018000233

**Published:** 2018-01-09

**Authors:** Palaniyappan Sivajeyanthi, Muthaiah Jeevaraj, Bellarmin Edison, Kasthuri Balasubramani

**Affiliations:** a Department of Chemistry, Government Arts College (Autonomous), Thanthonimalai, Karur- 639 005, Tamil Nadu, India

**Keywords:** crystal structure, Schiff base, hydrazinecarbo­thio­amide, hydrogen bonding, Hirshfeld surface analysis

## Abstract

The mol­ecule of the title Schiff base, C_8_H_8_BrN_3_OS·C_2_H_6_OS, which crystallizes as a di­methyl sulfoxide (DMSO) monosolvate, displays an *E* configuration with respect to the C=N bond, with a dihedral angle of 14.54 (11)° between the benzene ring and the mean plane of the N—N—C(N)=S unit. In the crystal, mol­ecules are linked by N—H⋯O hydrogen bonds, forming chains propagating along the *b*-axis direction. Within the chains there are *R* ^2^_3_(11) ring motifs, which are reinforced by C—H⋯O_DMSO_ hydrogen bonds enclosing secondary *R* ^1^_2_(6) and *R* ^2^_3_(9) loops. The chains are linked by O—H_hydrox­yl_⋯S hydrogen bonds, forming layers parallel to (011). Inversion-related layers are linked by short Br⋯Br inter­actions [3.5585 (5) Å], forming slabs parallel to (011). The inter­molecular inter­actions have been investigated using Hirshfeld surface studies and two-dimensional fingerprint plots. The crystal structure of the unsolvated form of the title compound has been reported previously [Kargar *et al.* (2010). Acta Cryst. E**66**, o2999], and its solid-state structure is compared with that of the title solvated form.

## Abstract 2

The mol­ecule of the title Schiff base, has an *E* conformation with respect to the C=N bond, and a dihedral angle of 14.54 (11)° between the benzene ring and the mean plane of the N—N—C(N)=S hydrazinecarbo­thio­amide unit.

## Chemical context   

Schiff bases are nitro­gen-containing active organic compounds that play a vital role in enzymatic reactions involving inter­action of an enzyme with a carbonyl group of a substrate (Tidwell, 2008 ▸). Thio­semicarbazones exhibit inter­esting pharmacological properties and biological activities. Thio­semicarbazone derivatives have gained special importance because of their role in drug development; for example they are used as anti­viral, anti­tubercular, anti-bacterial infection, analgesic and anti­allergic agents and in the treatment of central nervous system disorders and as sodium channel blockers and show anti­tumorous activity. The pharmacological versatility of semicarbazones, thio­semicarbazones and their metal complexes have been reviewed by Beraldo & Gambino (2004 ▸).

Thio­semicarbazones are formed by the condensation of thio­semicarbazides with aldehydes or ketones (Sriram *et al.*, 2006 ▸; Scovill *et al.*, 1982 ▸). They are also used in most branches of chemistry, for example, as dyes, photographic films, plastics and in the textile industry. These types of compounds also act as ligands for a variety of transition metals, often as high propensity multi-dentate chelating agents (Al-Karawi *et al.*, 2009 ▸). Herein, we report on the crystal structure of the title thio­semicarbazone that crystallizes as a dimethyl sulfoxide monosolvate. The crystal structure of the unsolvated form of the title Schiff base has been reported previously (Kargar *et al.*, 2010*a* ▸), and its solid-state structure is compared with that of the title solvated form.
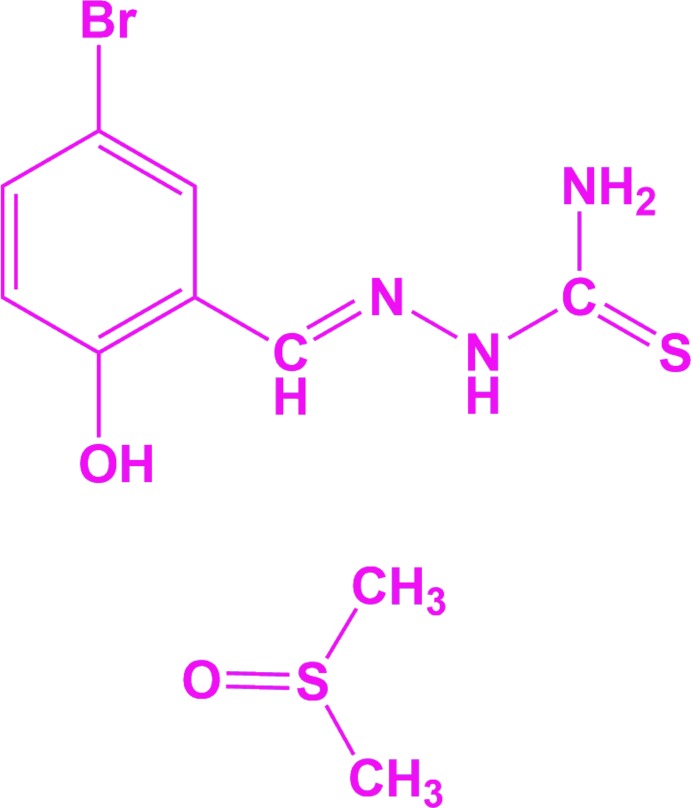


## Structural commentary   

**Figure 1:**
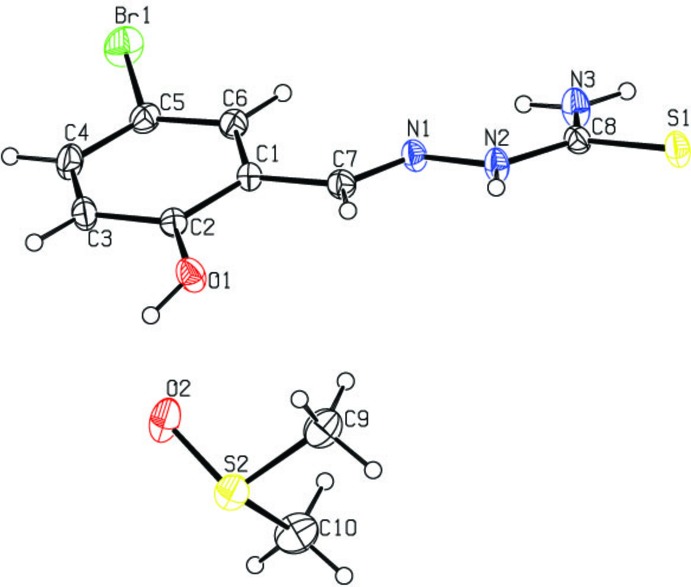
A view of the mol­ecular structure of the title compound, with the atom labelling. Displacement ellipsoids are drawn at the 50% probability level.

The mol­ecular structure of the title compound is shown in Fig. 1 ▸. The thio­semicarbazone mol­ecule has an *E* configurationabout the C7=N1 bond. The mol­ecule is twisted with a dihedral angle of 14.54 (11)° between the benzene ring and the mean plane of the N1/N2/C8/N3/S1 unit. The C8—-S1 bond distance of 1.698 (2) Å is close to that expected for a C=S bond (Cambridge Structural Database; Groom *et al.*, 2016 ▸). This confirms the existence of the compound in the thio­amido form in the solid state, similar to the situation observed in some related compounds, *viz*. (*E*)-2-(2,4-di­hydroxy­benzyl­idene)thio­semicarbazone and (*E*)-2-[(1*H*-indol-3-yl)methyl­ene]thio­semicarbazone (Yıldız *et al.*, 2009 ▸). The C1—N7 bond distance is 1.278 (3) Å, close to that of a C=N double bond, confirming the azomethine bond formation, again similar to the situation observed in related compounds, *viz*. (*E*)-1-[4-(di­methyl­amino)­benzyl­idene]thio­semicarbazide (Sun *et al.* 2009 ▸) and 2-[(2-hy­droxy­naphthalen-1-yl)methyl­ene]hydra­zine­carbo­thio­amide (Sivajeyanthi *et al.* 2017 ▸).

**Figure 2:**
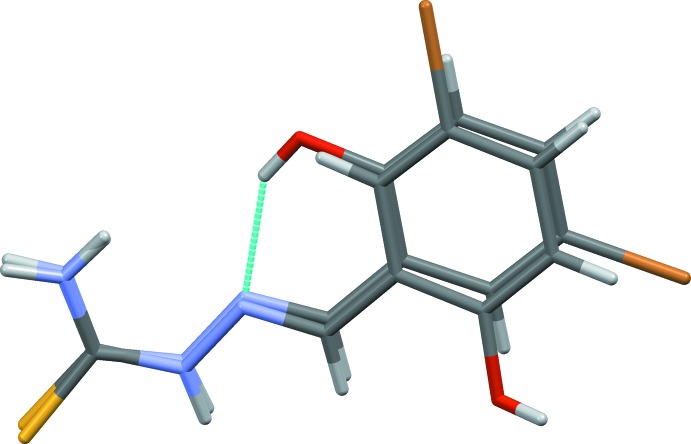
The structural overlay of the title mol­ecule with that of the unsolvated form (CEDPAE; Kargar *et al.*, 2010*a* ▸), showing the presence of the intra­molecular O—H⋯N hydrogen bond (dashed line) in CEDPAE.

In the mol­ecular structure of the unsolvated form of the title compound (Kargar *et al.*, 2010*a* ▸), an intra­molecular O—H⋯N hydrogen bond is present enclosing an *S*(6) ring motif. Comparing the two mol­ecules, as shown in the structural overlay of Fig. 2 ▸, it can be seen that the benzene ring of the title solvated compound is rotated by *ca*. 180° with respect to that in the unsolvated form of the mol­ecule. The bond lengths and bond angles of the two mol­ecules are similar. In the title compound, the dihedral angle between the benzene ring and the mean plane of the N—N—C(N)=S hydrazinecarbo­thio­amide unit is 14.54 (11)° compared to *ca* 7.05° in the unsolvated phase. Kargar *et al.* (2010*b* ▸) have also reported the crystal structure of the unsolvated chloro-substituted analogue. This mol­ecule has the same conformation as the unsolvated bromo-substituted analogue (Kargar *et al.*, 2010*a* ▸), but in contrast it crystallizes in the monoclinic space group *P*2_1_/*c*, while the unsolvated bromo compound crystallizes in the chiral ortho­rhom­bic space group *P*2_1_2_1_2_1_.

## Supra­molecular features   

**Figure 3:**
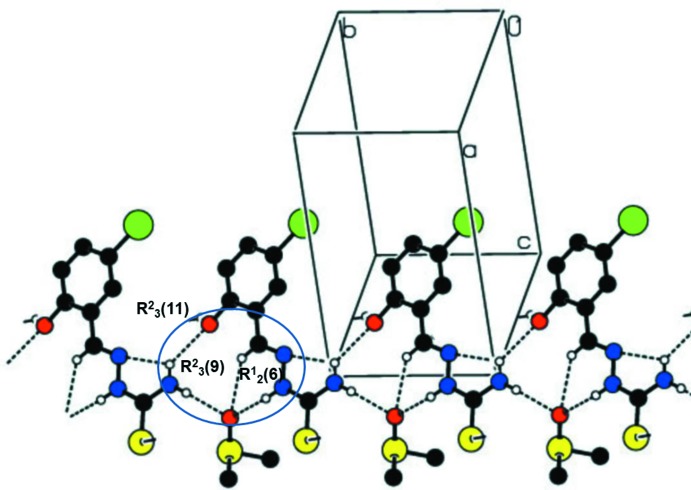
A partial view, almost normal to the (011) plane, of the hydrogen-bonded chain (dashed lines; see Table 1 ▸) propagating along the [010] direction. In this and subsequent figures, only the H atoms involved in hydrogen bonding have been included.

**Figure 4:**
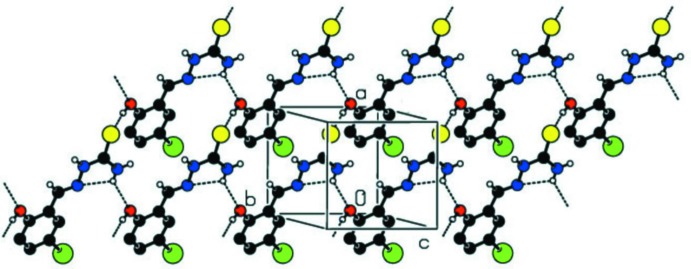
A view, almost normal to (011), of the hydrogen-bonded sheets parallel to (011). The hydrogen bonds are shown as dashed lines and details are given in Table 1 ▸.

**Figure 5:**
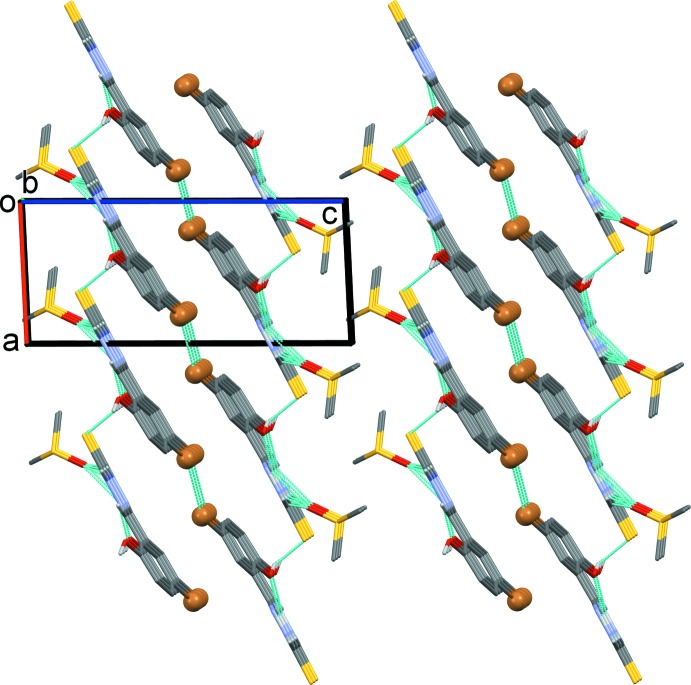
A view along the *b* axis of the crystal packing of the title compound. The hydrogen bonds (see Table 1 ▸) and the short Br⋯Br inter­actions are shown as dashed lines.

In the crystal, the Schiff base hydrazone is hydrogen bonded (see Table 1 ▸) to the di­methyl sulfoxide solvate mol­ecule, forming a chain propagating along the *b*-axis direction, as shown in Fig. 3 ▸. Within the chains there are 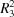
(11) ring motifs, which are reinforced by C—H⋯O_DMSO_ hydrogen bonds enclosing secondary 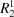
(6) and 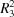
(9) ring motifs (Table 1 ▸). The 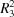
(11) ring motif is formed by N2—H2⋯O2^ii^, N3—H3*A*⋯O1^iii^ and N3—H3*B*⋯O2^iv^ hydrogen-bonding inter­actions, and the 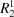
(6) ring motif is formed *via* C7—H7⋯O2^ii^ and N2—H2⋯O2^ii^ hydrogen-bonding inter­actions. Hence, atom O2 of the di­methyl sulfoxide acts as a trifurcated acceptor (Fig. 3 ▸, Table 1 ▸). The chains are linked by O1—H1⋯S^i^ hydrogen bonds, forming layers parallel to plane (011); see Fig. 4 ▸ and Table 1 ▸. Inversion-related layers are linked by short Br⋯Br(−*x*, −*y* + 1, −*z* + 1) inter­actions of 3.5585 (5) Å, forming slabs parallel to (011), as illustrated in Fig. 5 ▸.

## Hirshfeld surface analysis   

**Figure 6:**
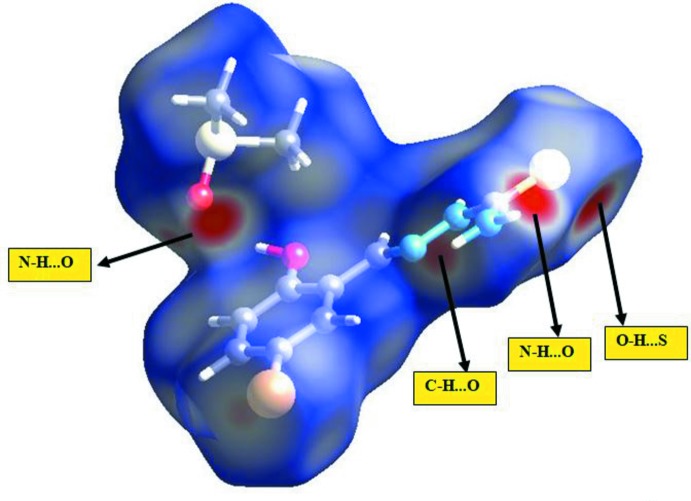
Hirshfeld surfaces mapped over *d*_norm_ for the title compound.

**Figure 7:**
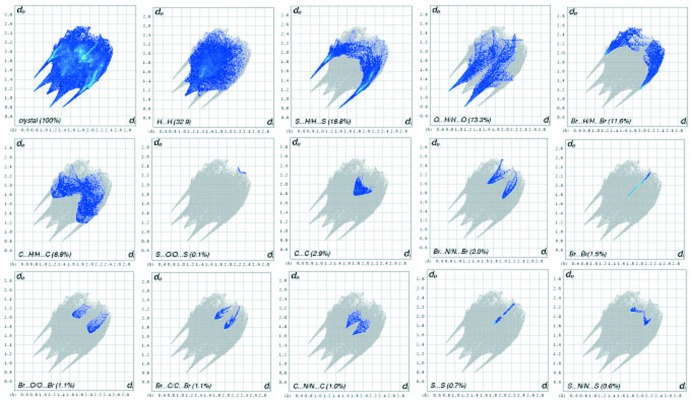
Two-dimensional fingerprint plots of the crystal and the relative contributions of the atom pairs to the Hirshfeld surface.

The three-dimensional *d*_norm_ surface is a useful tool to analyse and visualize the inter-mol­ecular inter­actions. *d*_norm_ takes negative or positive values depending on whether the inter­molecular contact is shorter or longer than the van der Waals radii (Spackman & Jayatilaka, 2009 ▸; McKinnon *et al.*, 2007 ▸). The three-dimensional *d*_norm_ surface of the title compound is shown in Fig. 6 ▸. The red points, which represent closer contacts and negative *d*_norm_ values on the surface, correspond to the N—H⋯O, O—H⋯S and C—H⋯O inter­actions. The percentage contributions of various contacts to the total Hirshfeld surface are as follows: H⋯H (32.9%), S⋯H/H⋯S (18.8%), O⋯H/H⋯O (13.3%), Br⋯H/H⋯Br (11.6), C⋯H/H⋯C (8.8%), N⋯H/H⋯N (3.4%), C⋯C (2.8%), Br⋯N/N⋯Br (2.0%), Br⋯Br (1.5%), Br⋯O/O⋯Br (1.1%), Br⋯C/C⋯Br (1.1%), C⋯N/N⋯C (1.0%), S⋯S (0.7%), S⋯N/N⋯S (0.6%) and S⋯C/C⋯S (0.2%), as shown in the two-dimensional fingerprint plots in Fig. 7 ▸.

## Database survey   

A search of the Cambridge Structural Database (Version 5.38, update May 2017; Groom *et al.*, 2016 ▸) for the 2-hy­droxy­benzaldehyde thio­semicarbazone skeleton (or salicyl­aldehyde thio­semicarbazone) yielded 25 hits. These include the unsolvated bromo- and chloro-substituted analogues of the title compound mentioned above, *viz*. 5-bromo-2-hy­droxy­benzaldehyde thio­semicarbazone (CEDPAE; Kargar *et al.*, 2010*a* ▸) and 2-(5-chloro-2-hy­droxy­benzyl­idene)hydra­zine­carbo­thio­amide (VACGUD; Kargar *et al.*, 2010*b* ▸). The crystal structure of salicyl­aldehyde thio­semicarbazone has also been reported at 295 K (GEXKID; Chattopadhyay *et al.*, 1988 ▸) and at 100 K (GEXKID01; Novaković *et al.*, 2007 ▸). The crystal structures of various hydrated forms of salicyl­aldehydethio­semicarbazone [(*E*)-2-(2-hy­droxy­benzyl­idene)hydrazine­carbo­thio­amide hydrate] have been reported at 100 K (UJIPIN) and 203 K (UJIPOT and UJIPUZ) by Monfared *et al.* (2010 ▸). In the majority of the hits, the 2-hy­droxy group forms an intra­molecular O—H⋯N hydrogen bond, as shown for CEDPAE in Fig. 2 ▸. Consequently, in the compounds mentioned above, the dihedral angle between the benzene ring and the mean plane of the N—N—C(N)=S hydrazinecarbo­thio­amide unit is relatively small, varying from *ca* 5.62 to 10.10°, compared to 14.54 (11)° in the title compound.

## Synthesis and crystallization   

The title compound was synthesized by refluxing for 8 h a 1:1 molar ratio of a hot ethano­lic solution (20 ml) of thio­semicarbazide (0.091 mg, Aldrich) and a hot ethano­lic solution of 5-bromo­salicyl­aldehyde (0.196 mg, Aldrich). The solution was then cooled and kept at room temperature. The precipitate that formed was filtered off and recrystallized from di­methyl sulfoxide. Colourless block-like crystals, suitable for the X-ray analysis, were obtained in a few days on slow evaporation of the solvent.

## Refinement   

Crystal data, data collection and structure refinement details are summarized in Table 2 ▸. The hydrogen atoms were fixed geometrically (O—H = 0.82, N—H = 0.86, C—H = 0.93–0.96 Å) and allowed to ride on their parent atoms with *U*_iso_(H) = 1.5*U*_eq_(O-hydroxyl, C-meth­yl) and 1.2*U*_eq_(N,C) for other H atoms.

## Supplementary Material

Crystal structure: contains datablock(s) global, I, 1. DOI: 10.1107/S2056989018000233/su5414sup1.cif

Structure factors: contains datablock(s) I. DOI: 10.1107/S2056989018000233/su5414Isup2.hkl

Supporting information file. DOI: 10.1107/S2056989018000233/su5414Isup3.cml

CCDC reference: 1587285

Additional supporting information:  crystallographic information; 3D view; checkCIF report

## Figures and Tables

**Table 1:** Hydrogen-bond geometry (Å, °)

*D*—H⋯*A*	*D*—H	H⋯*A*	*D*⋯*A*	*D*—H⋯*A*
O1—H1⋯S1^i^	0.82	2.40	3.1655 (17)	157
N2—H2⋯O2^ii^	0.86	2.10	2.897 (2)	155
N3—H3*A*⋯O1^iii^	0.86	2.37	3.048 (2)	136
N3—H3*B*⋯O2^iv^	0.86	2.11	2.930 (3)	160
C7—H7⋯O2^ii^	0.93	2.53	3.315 (3)	142

Symmetry codes: (i) 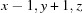; (ii) 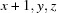; (iii) 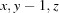; (iv) 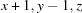.

**Table 2:** Experimental details

Crystal data	
Chemical formula	C_8_H_8_BrN_3_OS·C_2_H_6_OS
*M*_r_	352.26
Crystal system, space group	Triclinic, *P* 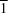
Temperature (K)	296
*a*, *b*, *c* (Å)	6.5411 (4), 7.3889 (6), 15.0662 (12)
α, β, γ (°)	78.772 (3), 86.872 (3), 87.376 (3)
*V* (Å^3^)	712.71 (9)
*Z*	2
Radiation type	Mo *K*α
μ (mm^−1^)	3.17
Crystal size (mm)	0.30 × 0.20 × 0.20

Data collection	
Diffractometer	Bruker Kappa APEXII CCD
Absorption correction	Multi-scan (*SADABS*; Bruker, 2004 ▸)
*T*_min_, *T*_max_	0.449, 0.569
No. of measured, independent and observed [*I* > 2σ(*I*)] reflections	6030, 3299, 2661
*R*_int_	0.018
(sin θ/λ)_max_ (Å^−1^)	0.666

Refinement	
*R*[*F* ^2^ > 2σ(*F* ^2^)], *wR*(*F* ^2^), *S*	0.030, 0.106, 0.79
No. of reflections	3299
No. of parameters	165
H-atom treatment	H-atom parameters constrained
Δρ_max_, Δρ_min_ (e Å^−3^)	0.27, −0.34

Computer programs: *APEX2*, *SAINT* and *XPREP* (Bruker, 2004 ▸), *SIR92* (Altomare *et al.*, 1993 ▸), *SHELXL2017/1* (Sheldrick, 2015 ▸), *PLATON* (Spek 2009 ▸), *Mercury* (Macrae *et al.*, 2008 ▸) and *publCIF* (Westrip, 2010 ▸).

## supplementary crystallographic information

### Crystal data

**Table:**

C_8_H_8_BrN_3_OS·C_2_H_6_OS	*Z* = 2
*M**_r_* = 352.26	*F*(000) = 356
Triclinic, *P*1	*D*_x_ = 1.637 Mg m^−^^3^
*a* = 6.5411 (4) Å	Mo *K*α radiation, λ = 0.71073 Å
*b* = 7.3889 (6) Å	Cell parameters from 2934 reflections
*c* = 15.0662 (12) Å	θ = 5.5–55.9°
α = 78.772 (3)°	µ = 3.17 mm^−^^1^
β = 86.872 (3)°	*T* = 296 K
γ = 87.376 (3)°	Block, colourless
*V* = 712.71 (9) Å^3^	0.30 × 0.20 × 0.20 mm

### Data collection

**Table:**

Bruker Kappa APEXII CCD diffractometer	2661 reflections with *I* > 2σ(*I*)
Radiation source: fine-focus sealed tube	*R*_int_ = 0.018
ω and φ scan	θ_max_ = 28.3°, θ_min_ = 3.4°
Absorption correction: multi-scan (SADABS; Bruker, 2004)	*h* = −8→6
*T*_min_ = 0.449, *T*_max_ = 0.569	*k* = −9→9
6030 measured reflections	*l* = −17→19
3299 independent reflections	

### Refinement

**Table:**

Refinement on *F*^2^	Primary atom site location: structure-invariant direct methods
Least-squares matrix: full	Secondary atom site location: difference Fourier map
*R*[*F*^2^ > 2σ(*F*^2^)] = 0.030	Hydrogen site location: inferred from neighbouring sites
*wR*(*F*^2^) = 0.106	H-atom parameters constrained
*S* = 0.79	*w* = 1/[σ^2^(*F*_o_^2^) + (0.1*P*)^2^] where *P* = (*F*_o_^2^ + 2*F*_c_^2^)/3
3299 reflections	(Δ/σ)_max_ = 0.001
165 parameters	Δρ_max_ = 0.27 e Å^−^^3^
0 restraints	Δρ_min_ = −0.34 e Å^−^^3^

### Special details

**Table:**

Geometry. Bond distances, angles etc. have been calculated using the rounded fractional coordinates. All su's are estimated from the variances of the (full) variance-covariance matrix. The cell esds are taken into account in the estimation of distances, angles and torsion angles
Refinement. Refinement of F^2^ against ALL reflections. The weighted R-factor wR and goodness of fit S are based on F^2^, conventional R-factors R are based on F, with F set to zero for negative F^2^. The threshold expression of F^2^ > 2sigma(F^2^) is used only for calculating R-factors(gt) etc. and is not relevant to the choice of reflections for refinement. R-factors based on F^2^ are statistically about twice as large as those based on F, and R- factors based on ALL data will be even larger.

### Fractional atomic coordinates and isotropic or equivalent isotropic displacement parameters (Å^2^)

**Table:**

	*x*	*y*	*z*	*U*_iso_*/*U*_eq_	
Br1	0.20262 (4)	0.63938 (4)	0.51812 (2)	0.0512 (1)	
S1	1.36501 (8)	0.37819 (8)	0.83370 (4)	0.0359 (2)	
S2	0.24643 (10)	0.89596 (8)	0.93448 (4)	0.0421 (2)	
O1	0.6312 (2)	1.1359 (2)	0.71559 (12)	0.0382 (5)	
N1	0.8705 (3)	0.6223 (3)	0.72520 (13)	0.0300 (5)	
N2	1.0553 (3)	0.5884 (2)	0.76701 (13)	0.0305 (5)	
N3	1.0303 (3)	0.2820 (3)	0.76421 (15)	0.0389 (6)	
C1	0.6169 (3)	0.8495 (3)	0.66806 (14)	0.0267 (6)	
O2	0.1487 (3)	0.9012 (2)	0.84564 (14)	0.0512 (6)	
C2	0.5325 (3)	1.0274 (3)	0.66902 (15)	0.0287 (6)	
C3	0.3559 (3)	1.0884 (3)	0.62389 (16)	0.0346 (7)	
C4	0.2574 (4)	0.9727 (3)	0.57957 (17)	0.0385 (7)	
C5	0.3383 (3)	0.7961 (3)	0.58003 (15)	0.0325 (6)	
C6	0.5159 (3)	0.7339 (3)	0.62232 (15)	0.0309 (6)	
C7	0.8054 (3)	0.7906 (3)	0.71317 (15)	0.0300 (6)	
C8	1.1348 (3)	0.4159 (3)	0.78513 (14)	0.0274 (6)	
C9	0.5074 (4)	0.8287 (4)	0.9172 (2)	0.0493 (9)	
C10	0.1671 (5)	0.6884 (4)	1.0067 (2)	0.0578 (10)	
H1	0.54961	1.21258	0.73121	0.0570*	
H2	1.11849	0.67747	0.78122	0.0360*	
H3	0.30338	1.20764	0.62341	0.0410*	
H3A	0.91417	0.30700	0.73958	0.0470*	
H3B	1.07844	0.17008	0.77525	0.0470*	
H4	0.13811	1.01311	0.54976	0.0460*	
H6	0.56908	0.61567	0.62069	0.0370*	
H7	0.88039	0.87680	0.73376	0.0360*	
H9A	0.51533	0.72024	0.89072	0.0740*	
H9B	0.57191	0.80243	0.97422	0.0740*	
H9C	0.57610	0.92693	0.87721	0.0740*	
H10A	0.02145	0.69522	1.01827	0.0870*	
H10B	0.23443	0.67244	1.06288	0.0870*	
H10C	0.20263	0.58551	0.97788	0.0870*	

### Atomic displacement parameters (Å^2^)

**Table:**

	*U*^11^	*U*^22^	*U*^33^	*U*^12^	*U*^13^	*U*^23^
Br1	0.0489 (2)	0.0567 (2)	0.0538 (2)	−0.0095 (1)	−0.0172 (1)	−0.0193 (1)
S1	0.0323 (3)	0.0315 (3)	0.0469 (4)	0.0088 (2)	−0.0164 (2)	−0.0132 (2)
S2	0.0571 (4)	0.0295 (3)	0.0432 (4)	−0.0032 (3)	−0.0125 (3)	−0.0124 (3)
O1	0.0344 (8)	0.0356 (8)	0.0498 (10)	0.0071 (7)	−0.0122 (7)	−0.0203 (7)
N1	0.0244 (8)	0.0301 (9)	0.0365 (10)	0.0043 (7)	−0.0081 (7)	−0.0086 (7)
N2	0.0273 (9)	0.0241 (8)	0.0417 (11)	0.0023 (7)	−0.0132 (7)	−0.0082 (7)
N3	0.0374 (10)	0.0260 (9)	0.0566 (14)	0.0019 (8)	−0.0182 (9)	−0.0123 (9)
C1	0.0254 (10)	0.0273 (10)	0.0267 (10)	0.0031 (8)	−0.0038 (7)	−0.0040 (8)
O2	0.0724 (13)	0.0296 (8)	0.0554 (12)	0.0027 (8)	−0.0339 (10)	−0.0099 (8)
C2	0.0294 (10)	0.0280 (10)	0.0288 (11)	0.0017 (8)	−0.0032 (8)	−0.0057 (8)
C3	0.0341 (11)	0.0302 (11)	0.0390 (13)	0.0091 (9)	−0.0089 (9)	−0.0059 (9)
C4	0.0303 (11)	0.0460 (13)	0.0373 (13)	0.0063 (10)	−0.0115 (9)	−0.0024 (10)
C5	0.0314 (10)	0.0377 (11)	0.0289 (11)	−0.0033 (9)	−0.0054 (8)	−0.0063 (9)
C6	0.0328 (11)	0.0306 (10)	0.0297 (11)	0.0012 (8)	−0.0047 (8)	−0.0063 (9)
C7	0.0306 (11)	0.0290 (10)	0.0314 (11)	0.0008 (8)	−0.0039 (8)	−0.0084 (8)
C8	0.0283 (10)	0.0249 (10)	0.0301 (11)	0.0030 (8)	−0.0031 (8)	−0.0083 (8)
C9	0.0524 (16)	0.0504 (15)	0.0462 (16)	−0.0097 (12)	−0.0098 (12)	−0.0079 (12)
C10	0.0619 (18)	0.0567 (17)	0.0521 (18)	−0.0143 (14)	0.0035 (13)	−0.0025 (14)

### Geometric parameters (Å, º)

**Table:**

Br1—C5	1.898 (2)	N3—H3B	0.8600
S1—C8	1.698 (2)	C3—C4	1.383 (3)
S2—C10	1.780 (3)	N3—H3A	0.8600
S2—O2	1.508 (2)	C4—C5	1.384 (3)
S2—C9	1.776 (3)	C5—C6	1.373 (3)
O1—C2	1.367 (3)	C3—H3	0.9300
N1—N2	1.384 (3)	C4—H4	0.9300
N1—C7	1.278 (3)	C6—H6	0.9300
O1—H1	0.8200	C7—H7	0.9300
N2—C8	1.338 (3)	C9—H9A	0.9600
N3—C8	1.324 (3)	C9—H9B	0.9600
C1—C7	1.449 (3)	C9—H9C	0.9600
C1—C2	1.405 (3)	C10—H10A	0.9600
C1—C6	1.404 (3)	C10—H10B	0.9600
C2—C3	1.385 (3)	C10—H10C	0.9600
N2—H2	0.8600		
			
C9—S2—C10	97.89 (14)	S1—C8—N2	118.69 (16)
O2—S2—C9	105.88 (12)	N2—C8—N3	118.41 (19)
O2—S2—C10	105.84 (12)	S1—C8—N3	122.90 (18)
N2—N1—C7	114.45 (19)	C2—C3—H3	120.00
C2—O1—H1	110.00	C4—C3—H3	120.00
N1—N2—C8	119.44 (18)	C5—C4—H4	120.00
C2—C1—C7	119.58 (19)	C3—C4—H4	120.00
C2—C1—C6	118.62 (19)	C1—C6—H6	120.00
C6—C1—C7	121.8 (2)	C5—C6—H6	120.00
O1—C2—C3	122.0 (2)	N1—C7—H7	119.00
C1—C2—C3	120.5 (2)	C1—C7—H7	119.00
N1—N2—H2	120.00	S2—C9—H9A	109.00
C8—N2—H2	120.00	S2—C9—H9B	110.00
O1—C2—C1	117.53 (18)	S2—C9—H9C	109.00
H3A—N3—H3B	120.00	H9A—C9—H9B	109.00
C2—C3—C4	120.3 (2)	H9A—C9—H9C	109.00
C8—N3—H3B	120.00	H9B—C9—H9C	109.00
C8—N3—H3A	120.00	S2—C10—H10A	109.00
C3—C4—C5	119.3 (2)	S2—C10—H10B	109.00
Br1—C5—C6	119.57 (17)	S2—C10—H10C	109.00
Br1—C5—C4	118.85 (17)	H10A—C10—H10B	109.00
C4—C5—C6	121.6 (2)	H10A—C10—H10C	109.00
C1—C6—C5	119.8 (2)	H10B—C10—H10C	109.00
N1—C7—C1	121.2 (2)		
			
C7—N1—N2—C8	178.2 (2)	C2—C1—C7—N1	169.6 (2)
N2—N1—C7—C1	178.29 (19)	C6—C1—C7—N1	−10.9 (3)
N1—N2—C8—S1	178.26 (15)	O1—C2—C3—C4	−178.3 (2)
N1—N2—C8—N3	−1.7 (3)	C1—C2—C3—C4	1.9 (3)
C6—C1—C2—O1	178.65 (19)	C2—C3—C4—C5	−0.7 (4)
C6—C1—C2—C3	−1.5 (3)	C3—C4—C5—Br1	−179.52 (18)
C7—C1—C2—O1	−1.9 (3)	C3—C4—C5—C6	−1.0 (4)
C7—C1—C2—C3	178.0 (2)	Br1—C5—C6—C1	179.90 (16)
C2—C1—C6—C5	−0.1 (3)	C4—C5—C6—C1	1.4 (3)
C7—C1—C6—C5	−179.6 (2)		

### Hydrogen-bond geometry (Å, º)

**Table:**

*D*—H···*A*	*D*—H	H···*A*	*D*···*A*	*D*—H···*A*
O1—H1···S1^i^	0.82	2.40	3.1655 (17)	157
N2—H2···O2^ii^	0.86	2.10	2.897 (2)	155
N3—H3*A*···O1^iii^	0.86	2.37	3.048 (2)	136
N3—H3*A*···N1	0.86	2.30	2.648 (3)	104
N3—H3*B*···O2^iv^	0.86	2.11	2.930 (3)	160
C7—H7···O1	0.93	2.44	2.752 (3)	100
C7—H7···O2^ii^	0.93	2.53	3.315 (3)	142

Symmetry codes: (i) *x*−1, *y*+1, *z*; (ii) *x*+1, *y*, *z*; (iii) *x*, *y*−1, *z*; (iv) *x*+1, *y*−1, *z*.
